# Environmental impacts of shore revetment

**DOI:** 10.1016/j.heliyon.2023.e19646

**Published:** 2023-09-04

**Authors:** Sarinya Sanitwong-Na-Ayutthaya, Cherdvong Saengsupavanich, Effi Helmy Ariffin, Amila Sandaruwan Ratnayake, Lee Shin Yun

**Affiliations:** a Faculty of International Maritime Studies, Kasetsart University, Sri Racha Campus, Chonburi, Thailand; b Institute of Oceanography and Environment, Universiti Malaysia Terengganu, Terengganu, Malaysia; c Faculty of Science and Marine Environment, Universiti Malaysia Terengganu, Terengganu, Malaysia; d Faculty of Applied Sciences, Uva Wellassa University, Sri Lanka

**Keywords:** Environmental impacts, Shore protection structure, Beach environment, Coastal management, Sustainable coastal development

## Abstract

Coastal structures, especially revetments, have been widely implemented to protect properties and infrastructures from erosive waves during storms. While being incompatible with nature-based solutions, revetments have still been constructed due to their effectiveness in solving coastal erosion. One of the most crucial concerns that should be considered as part of a revetment implementation is how to diminish and manage its possible impacts on the environment. Thus, a thorough understanding of how the revetments affect the surrounding environment must be achieved. This article critically reviews and summarizes their economic considerations, and environmental impacts on beach morphology, hydrodynamics, ecology, aesthetics, beach accessibility, beach recreation, and other notable aspects. Coastal practitioners and researchers, who are involved with the revetments, may increase their environmental awareness before implementing them. The revetments can be an excellent option to protect the eroding shoreline, if their possible environmental consequences are well-understood and properly managed.

## 1 Introduction

Coastal defense structures have been implemented to solve coastal erosion and re-establish eroded beaches. Revetments, seawalls, and breakwaters are often installed to stabilize the shoreline [1,2]. The revetments are sloping-front engineering structures that have been implemented since ancient times. Coastal Engineering Manual summarizes that the revetments have been used by humans to protect vulnerable coastal stripes since 27 B.C., as evidenced in a classic treatise by Vitruvius, as well as Greek and Latin literature by Herodotus, Josephs, Suetonius, Pliny, Appian, Polibus, Strabo, and others that provided limited descriptions of the ancient coastal works [3]. Nowadays, they have been extensively constructed in coastal zones since the 1990s in numerous countries [[4], [5], [6], [7], [8]], especially Asian nations such as Japan [9], China [10], Thailand [11,12], Malaysia [2,13], and Indonesia [14]. Besides preventing coastal erosion, in certain cases where the revetments were carefully designed, they can also provide secondary functions such as parking area, facilitated beach access, and enhanced local coastal tourism [15,16].

Revetments differ in size, cost, durability, effectiveness, sustainability, and socio-environmental impacts. Over the past decade, concerns about the revetments have evolved because of their direct and indirect impacts. Although the revetments’ success in preventing shoreline retreat and enhancing urban coastal landscape has been well-acknowledged, they are often reported to degrade natural coastal habitats [17]. They can significantly alter coastal systems and may adversely impact both the project site and neighboring locations [18]. Since it is expected that implementing the revetments will be extensively needed to protect coastal communities in response to drastic climate change [19], there is a need to increase awareness and carefully assess environmental impacts [20,21].

**Fig. 1:**
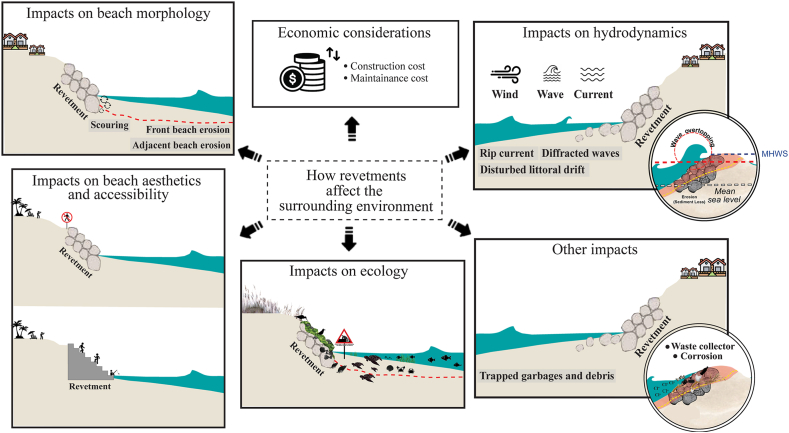
An interconnectedness of environmental impacts in this study.

Although revetments can promote the local community's well-being, they also induce physical and ecological changes [22]. In order to avoid or minimize the associated consequences, we provide the first review of how the revetments affect the surrounding environments by compiling key findings from published studies and highlighting their impacts. Besides a revetment's economical consideration, we also categorize different types of impacts or effects that are usually identified with the placement of the coastal revetments, such as beach morphology, hydrodynamics, ecology, beach aesthetics and accessibility, and other conspicuous consequences (Fig. 1). Our review article goes beyond a normal consideration of local-scale effects of the revetments because we also suggest potential ways to address such impacts and to critically identify research gaps that need to be filled. Hence, this article will be beneficial for coastal practitioners or researchers, who are interested in applying the revetments for coastal protection, to prepare and properly manage the foreseeable environmental consequences.

## 2 Revetments’ functions

**Fig. 2:**
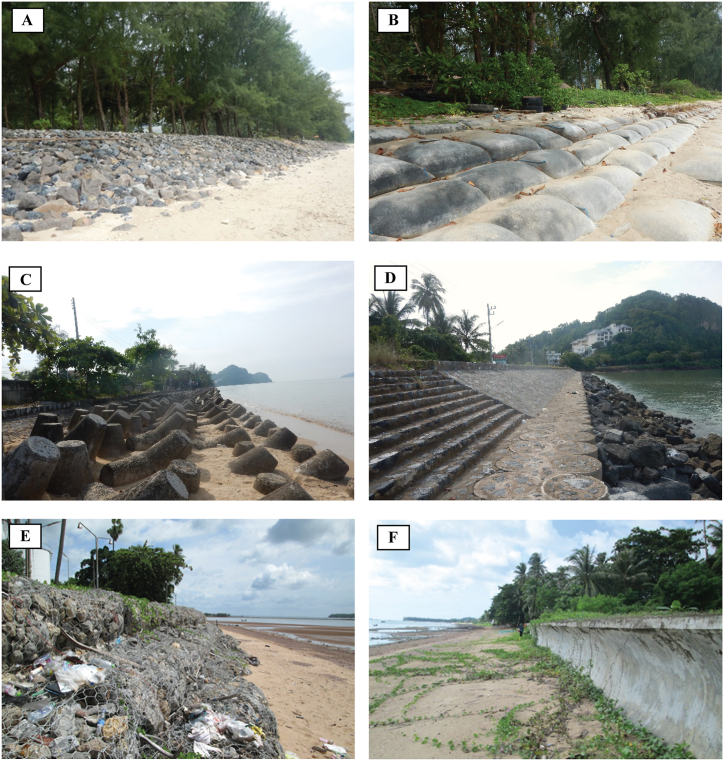
Different types of revetment. (A) A rock revetment, (B) a sandbag revetment, (C) a concrete tetrapod revetment, (D) a grouted rock revetment, (E) a gabion revetment, and (F) a curve-faced concrete revetment.

Revetments are sloping shore-parallel structures constructed on the beach to dissipate and reduce wave forces attacking a boundary between the sea and the land [23]. Revetment implementation is one of the most effective, prominent, and quickest solutions for shoreline erosion control. Unlike soft options which can take years to prove their unsuccessfulness, the revetments can guarantee that the shoreline is immediately stabilized and local communities can further flourish [16]. There is a large variety of revetment types (Fig. 2) designed for specific purposes, including rock revetments (Fig. 2A), sandbag revetments (Fig. 2B), concrete-unit revetments (Fig. 2C), grouted rock revetments (Fig. 2D), gabion revetments (Fig. 2E), curve-faced concrete revetments (Fig. 2F), and other materials such as polyurethane-bonded aggregates [24]. The amour layer of the coastal revetments can be either permeable or impermeable [25]. In some particular situations, the revetments can mimic the appearance and function of natural landforms by working together with other coastal protection measures, including breakwaters, groins, beach nourishment, and dikes [23]. However, some coastal practitioners may consider them one of the most expensive approaches that also cause adverse impacts on the surrounding coastal environment [2]. Thus, economic consideration and related environmental impacts are two of the necessary criteria for decision makers to judge whether the revetments are an appropriate approach to protect the coastline.

## 3 Economic consideration

Revetments are economically viable protection structures. Reducing the overall construction and maintenance costs spent over the revetment's lifetime is a goal of a cost-effective shore protection method. Several studies have shifted their focus from physical efficacy to more comprehensive coastal zone management by using economic tools such as cost-effectiveness, benefit-cost ratio, and efficiency evaluation of the coastal structures [26,27]. The most noticeable benefit of revetments is their ability to reduce physical damage to properties and infrastructures, and to increase economic outputs, if they are associated with tourists or other economic activities, [28]. Reference [29]; who carried out the cost-benefit analysis on the longitudinal revetments, concluded that they had better physical and economic performances than the shorter ones because they can economically break-even after thirteen years. A similar study conducted by Ref. [30] showed that revetments would receive the benefits from the avoided overtopping impacts, and break-even with the intervention costs in ten years. Therefore, the high net present value and the benefit-cost ratio, or the attractive break-even time can be a powerful driving motivation of selecting the revetments as the appropriate coastal protection method [31].

Construction materials for revetments influence their economic effectiveness. Many researchers confirmed that the revetments exhibit an economic advantage. The revetments built from rocks have the lowest annual and moderate construction costs because of the locally available materials and construction plants [32]. Nevertheless, an adequate quantity of rocks with appropriate properties is unavailable in some countries such as the Netherlands, Singapore, and Kuwait. Besides the construction cost, the technical study of [28] also proved that the revetments constructed from rocks and concrete armor units were very effective in protecting the land and typically had a long-lasting life. It was further proved by Ref. [33]; who stated that the rock materials of revetments had the least reported failures. In addition, a comparison study on the economic value of rock revetments and mangroves conducted by Ref. [8] concluded that both could function effectively to protect the coastline, but rock revetments would require less land, although they needed more upfront construction costs for risk reduction and adaptation when compared to the mangroves. Based on behavioral economics, willingness-to-pay plays an essential element in advocating that the revetments are welcome by local residents. In Vietnam, the economic analysis from the study by Nyugen et al. (2021) showed that the people agreed to provide greater value for public beaches with trees and restaurants, protected by visible structures such as stair revetments to prevent further erosion. Simultaneously, they were also willing to pay a local tax to fund the erosion protection program in order to increase the local economy and recreational activities.

From an economic perspective, maintenance and repair costs of revetments can be crucial for coastal property owners or governments in deciding how to protect the eroding shoreline. For example, implementing regular maintenance can help prevent costly repairs of the revetment and ensure that they always work efficiently. The revetments have been increasingly criticized due to their high investment and maintenance costs as they are frequently exposed to severe environmental conditions [34,35]. Revetment rocks require periodic maintenance by adding new rocks every five to ten years because the toe rocks may emerge above sand if no extra action (e.g., nourishment) is taken [36]. It is also supported by a recent study of [4]; who stated that revetments need long-term maintenance and high repair costs because of their high deterioration rate. Reference [37] mentioned that the high deterioration rate was due to cracks on the armor units of the revetments which caused them to break up, demanding an entire replacement after only 22 years, instead of withstanding until 50 years of its designed life. References [5,33] summarized that certain types of revetment required intensive maintenance throughout their service lifespans because some construction materials (e.g. riprap rocks, polyurethane-bonded aggregates, or geotextile sandbags) were not durable, and needed to be regularly rearranged or augmented as they could be easily displaced and destroyed during storm events.

## 4 Impact on beach morphology

**Fig. 3:**
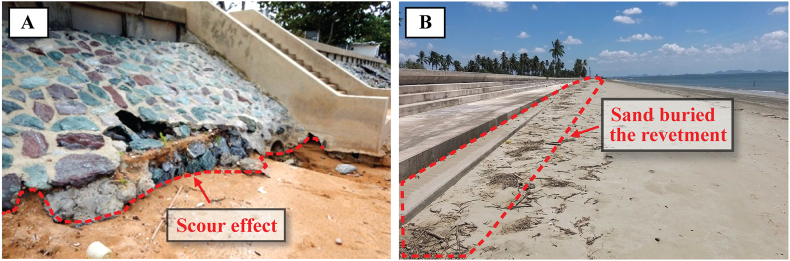
Front beach of revetment (A) toe scouring, (B) sand climbing on the front slope.

**Fig. 4:**
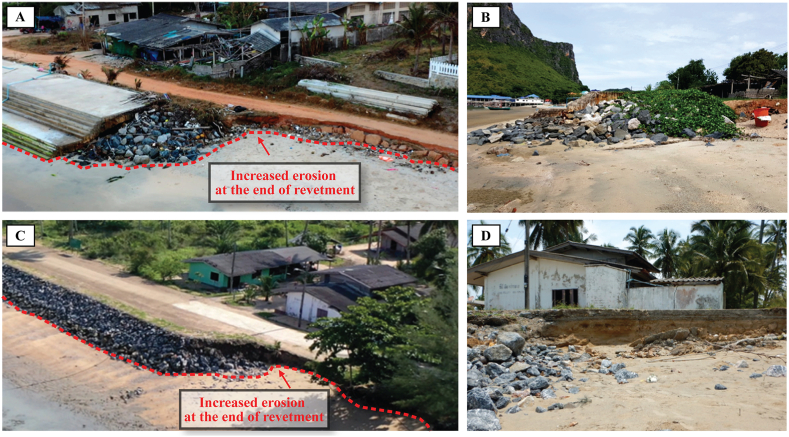
Adjacent beach morphology with revetment. (A, B) Flanking effect at the downdrift end of the stepped revetment. (C, D) Flanking effect at the downdrift end of the rock revetment.

Revetments significantly affect beach morphology (e.g., beach width, coastline shape, beach volume), depending on water level, wave climate, and sediment supply. Examples of the impact on front beach morphology can be seen in Fig. 3, including scouring and sand burial. Waves, that hit the revetment face, break on and partly reflect from the structure. If the revetment surface is highly porous, less wave reflection will occur. Impermeable revetment surface increases the reflected waves, together with increasing intensity of backwash, the revetment toe will be scoured (Fig. 3A). On the other hand, during calm periods, waves can carry sediment and deposit it upon the revetment surface, if the revetment slope is not steep (Fig. 3B). Moreover, neighboring beaches can be affected by exacerbated erosion and flanking (Fig. 4) because waves that hit the revetment's tips diffract to an adjacent beach. Although the revetments do not intercept as much alongshore sediment transport as groins, they can induce downdrift erosion [38]. Both reasons make the location closest to the endpoint of the revetment the critical zone (Fig. 4A–D). Since coastal scientists and engineers have debated regarding the revetments' impacts on the fronting and adjacent beaches, the critical reviews in this section are divided into two sub-sections.

### 4.1 Impact on front beach morphology

Revetments are conventionally applied to protect beach-front properties by limiting wave run-up from overtopping the buildings [39]. Reference [40] revealed that the revetment essentially served as a scarp toe protection, dissipating swash flows and preventing moderate wave conditions reflecting from the steep scarp face. Despite the fact that the revetments are highly effective in solving coastal erosion, they are likely to induce scouring in front of them [41,42]. The scouring of the front beach berm, due to reflected waves, caused the elevation of the front beach to be lowered rapidly and made the revetments collapse more easily [43]. On the other hand, sediment, that used to be on the front beach, was carried further offshore by the reflected waves, shallowing the surf zone, dissipating more incoming wave energy due to a wave-breaking process, thus providing positive feedback for coastal protection. Reference [44] who evaluated and monitored the shorelines with rock revetment, highlighted that placing the rock revetments on the front beaches of Jekyll Island State Park protected the residential areas against storm surges. It was further evaluated that the existence of revetments had put the sand-sharing system dangerously out of balance.

Revetments have altered the front beach morphology, thereby changing the morphological behavior. Reference [45] argued that sloping rock revetments created less environmental impact than vertical concrete seawalls because the rocks could absorb rather than reflect the wave energy. Waves penetrated into rock voids are not reflected and it is an ideal approach for coastal protection. Reference [34] claimed that the dense pack of revetment could act as a concrete wall, causing the fronting beach to either narrow or disappear. In Italy, front beaches gradually became narrower after the implementation of revetments, and eventually disappeared altogether because they were experiencing a deficit in sediment supplies from rivers [46]. This conclusion was also supported by the findings of [18,45,47]; who mentioned that the fronting beach would be lost because of wave interactions with the revetments. The revetments can interrupt local sediment balance, whiles waves hitting the revetment are reflected downwards, scouring the toe of the revetment [48]. In addition, Ref. [49] concluded that the revetments had exacerbated erosion problems in Hermenegildo. Although the properties were protected by the revetments, the beach berm width was decreased, increasing the risk of structural damage because they were built too close to the sea without proper design and maintenance. On the contrary, Ref. [16] argued that the beach steepening should not be considered too significant, compared to other benefits that can be gained by the erosion-safe beach. If coastal decision makers overconcern with the fronting beach slope and let the erosion continue, there will eventually be no beach dune, no coastal infrastructures, no coastal tourism, and even no people living on the coastline.

### 4.2 Impact on adjacent beach morphology

Revetments can trigger updrift and downdrift beach changes because of the blockage of longshore sediment transport. Studies by Refs. [50,51] confirmed that the revetments could induce updrift accretion and downdrift erosion on adjacent beaches. Although most revetments are intended to halt beach erosion, it is worth noting that they can also erode the adjacent beach in some instances [13,52,53]. Firstly, a flanking phenomenon as an obvious negative impact has been realized and commonly occurred at the downdrift end of the revetments [46]. Secondly, many previous research reported that the revetments intercepted the longshore sediment transport, inducing downdrift erosion [[54], [55], [56]], but the magnitude of such downdrift erosion was not as severe as those of jetty or offshore breakwaters. The revetments may partly intercept alongshore sediment transport because their slopes protrude into the swash zone. Normally, the revetments are placed outside land deeds, where the erosion has already occurred. A tip of the revetments, constructed outside the deed to reclaim the eroded land, is similar to a small groin, inducing the downdrift erosion. Implementation of the revetments caused the downdrift beach berm to be narrower and could devastate nearby coastal properties [57]. Thirdly, in cases where revetments are constructed downdrift next to jetties or offshore breakwaters, the revetment will postpone the erosion further downdrift. Reference [57] presented that the jetty at Cha-Am beach, Thailand, created a downdrift erosion, and the revetments were constructed to solve it. Although the revetments could protect the beach it intended, the erosion was postponed further downdrift to the revetment's endpoint. Similarly, In Malaysia, a revetment was constructed after a series of offshore breakwaters along the Tok Jembal beach, Terengganu, causing the beach erosion to shift and moved further northwards, inducing adverse impacts on the adjacent unprotected beach [58,59]. Similar impacts were also highlighted by Refs. [60,61]. It may be concluded that, although the revetments could protect the properties behind them, they might reduce the amount of sand provided downdrift. However, the downdrift erosion will not happen if the endpoint of the revetments is located at proper locations such as headlands or river mouths. Reference [16] reported that the downdrift beach erosion could benefit a nearby creek, which would otherwise be clogged due to sediment deposition, facilitating inland water discharge and artisanal fishermen. Reference [12] reported that the revetment at the Laem Ngoo beach, Thailand, did not cause any downdrift erosion because it was constructed between two headlands.

## 5 Impact on hydrodynamics and structural integrity

Mild sloping revetments are known to dissipate waves, reducing hydrodynamics severity. Waves are forced to break upon a revetment slope. On the other hand, vertical or steep revetments induce strong wave reflection, increasing wave heights in front of them, potentially leading to scouring, and eventually undermining the revetments themselves. One of the most common hydrodynamics effects is wave overtopping, when wave uprush flows over a revetment's crest. Basic coastal engineering theory suggests that the rate of overtopping for a gentle slope would decrease quickly as the water depth in front of the revetment became shallow, because of the depth-limited wave breaking (US Army Corps of Engineers, 2002; [62]). If the revetment crest is too low, the overtopping can damage inland infrastructures. Many researchers concluded that the revetments could serve as a buffer against an excessive wave run-up [63], but sometimes they could be damaged (Table 1). In the case of rock revetments, the inadequate weight of rocks would cause the revetment to collapse during extreme events, contributing to sped-up erosion because the water could overtop the structures [34].

**Table 1:** A summary of revetment damage due to wave run-up and overtopping.

Study area	Consequences	References
North Carolina	Bulkhead or similar vertical-faced concrete revetments result in large amount of wave overtopping. Bulkhead maintenance often includes backfilling and repairment of revetment crest.	[6]
South Carolina	Most of the revetments in the study area were overtopped by storm surges and waves, causing 24% of revetments to be destroyed, 68% damaged	[64]
North Carolina	Hurricane Irene (in 2011), and Arthur (in 2014) damaged bulkheads or vertical-faced concrete revetments. The bulkheads are not living up to the expectation of superior durability or effectiveness during hurricanes, and are more costly to maintain than ripraps.	[63]
Shizuoka, Japan	Typhoon Hagibis's intensive landfall generated the highest-level storm surges at the head of Tokyo Bay. The high water levels predominantly caused by energetic swells were comparable with the crest heights of defense structures. The intensive typhoon did not trigger catastrophic damage, but caused minor flooding due to wave overtopping.	[65]

There are many options to enhance a reduction in wave overtopping, thus increasing the revetment's overall stability and effectiveness. In California, an integrated shore protective revetment with a pedestrian walkway had successfully provided additional protection against storm wave impingement, and the beach had recovered and exhibited a typical summer beach condition during the past El Nino seasons in 2009–2010 [66]. Alternatively, an application of engineering materials can further reduce hydrodynamics forces. The use of plastic filters on rubble revetments and interlocking block revetments have been applied, allowing the free flow of water to seep through the joints of blocks, strengthening the structures to survive wave attacks. A careful engineering design of the revetment crest by installing a parapet could effectively reduce the overtopping discharge, solving the abovementioned problem [62]. Reference [67] reported three successful cases of protecting Thailand beaches with stepped concrete revetment equipped with crest parapets.

Revetments intervene local hydro-dynamics (e.g., wave-current interactions) and cause a complex flow over and through them. The revetments have been proven to be dynamic because rocks could move and respond to wave forces [60,68]. According to Ref. [40]; the revetments could quickly adapt to changing wave conditions, reaching a stable profile after high tides, varying around a quasi-equilibrium state. Reference [69] showed that the revetments could divert longshore water currents and diffracted waves. Reference [60] concluded similar findings that the revetments on India's Manakudi, Putthandurai, and Midalam coasts disturbed the wave directions, altering local water current characteristics. The seaward movement of rip currents eroded beach materials and caused coastal land losses. In addition, Do et al. (2022) presented a time series of revetment photographs at Hujeong Beach, Korea, showing that the revetment could aggravate wave reflection, which resulted in the steepened front beach.

## 6 Impact on ecology

**Fig. 5:**
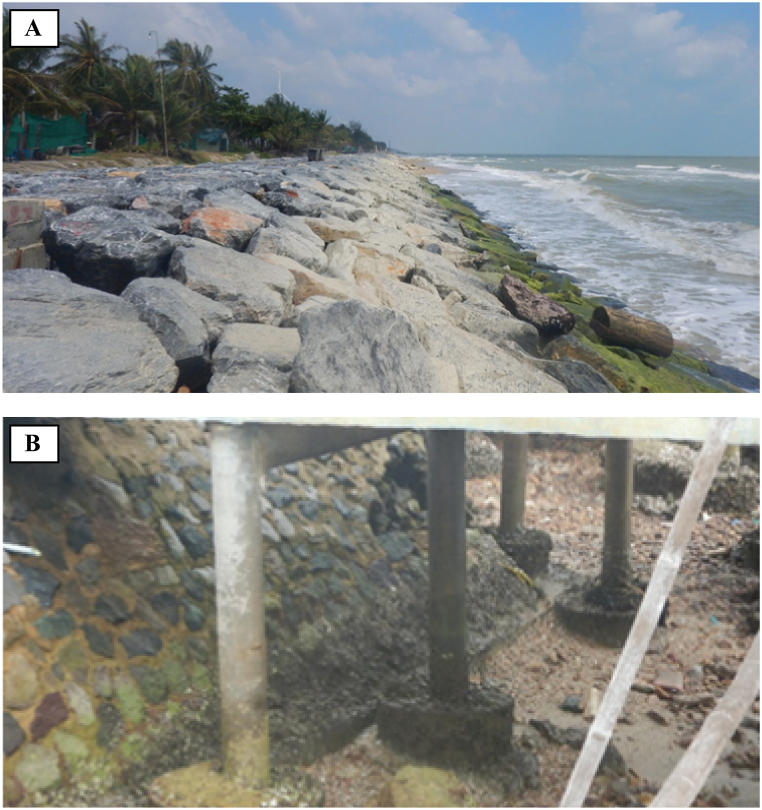
A presence of benthic epifauna on revetment surface. (A) A rock revetment, and (B) a concrete revetment.

Revetments not only have the potential to alter marine and coastal ecosystems at the sites of their installations, but they may also generate different negative or positive ecological impacts on the coastal environment. Fig. 5 reveals the presence of benthic epifauna on the revetment surface, resulting in alternative marine habitats. Marine organisms can attach to rock surfaces (Fig. 5A), and concrete surfaces (Fig. 5B), which emerge during low tides and submerged during high tides. These circumstances may change ecological settings. However, there is no robust conclusion that such changes in marine ecosystems due to the revetments are actually a benefit or a deterioration. Coastal and ecological scientists only know that there will be some kinds of ecological alternation, but cannot find any evidence to prove how they really promote or demote the environment. There are disputes on the effects of revetments on marine organisms and their surrounding environment, as the revetment may affect biodiversity and distribution patterns of benthic macrofauna, macroflora, and pelagic organisms in marine habitats. In this section, we review and summarize significant findings, concerning benthic community, fish assemblages, dune plants, bird community, and sea turtles.

### 6.1 Impact on benthic community

The presence of revetments can affect benthic, vertebrate, and invertebrate communities. Reference [70] proved that the benthic organisms (e.g., barnacles) were dominantly discovered and significantly enhanced on the surface of mid-tide rock revetment. Additional artificial rock pools on the rock revetments in this study facilitated the colonization of native marine species without any presence of an invasive species on the surface of the rock revetment. Biodiversity could be promoted on the rock revetments, which could be incorporated during the design phase. The ecological study by Ref. [71] stated that riprap revetments had greater benthos diversity and bivalve prey abundance in sub-tidal habitats, however, a density of macrobenthos (e.g., crabs) was significantly lower in the riprap revetments because of a reduction in food sources, and habitat suitability for these benthic organisms. Reference [72] found that sessile macroinvertebrates (e.g., sponges, corals, and tunicates) and macroalgae cover were higher in the riprap revetments than in natural mangrove areas because there were essential food sources for herbivorous fish.

The ecological impacts of revetment vary according to the nature of the surrounding habitats during the installation and maintenance phases. Reference [73] found that major intertidal benthic species (e.g., limpets, fucoid seaweeds, barnacles) had successfully colonized the rock revetments within 18 months of installation, proving that more time was required to re-establish the baseline species richness. Although the revetments are steeper and subjected to the minor tidal excursion, it was concluded that the rock revetments could produce a similar biotope as the natural shore platform to support quicker succession and high species density. Reference [74] reported that the intertidal species on the top surface of a rock revetment were challenged by the ecological disturbances from episodic revetment maintenance, which reduced suitability to dwell. In Japan, maintenance of a revetment on the coast of Kitsunezaki had negatively affected the benthic algae and kelp species, especially *Eisenia bicyclis* which was squashed and buried by large rocks [75]. It was further highlighted that the density of the kelp population near the revetments appeared to be unchanged, but a ratio of young plants appeared to decrease, and the population was skewed towards older plants. The disturbances generated by the revetment maintenance works reduced the diversity of epibiotic algae because it repeatedly reset the colonization.

### 6.2 Impact on fish assemblages

Revetments have a great potential to provide habitats for fish and crustaceans, in terms of access to food resources, although such a positive effect is less evident than that of breakwaters [76]. Riprap revetments have higher fish abundance and diversity of fish and crustaceans than bulkheads because ripraps provide more complex structural habitats than vertical bulkhead walls [77]. They also suggested that natural habitats and the riprap revetments in their study had similar fish community integrity, because they imitated natural shorelines with fissures and hard substrates. Reference [78] pointed out that seven fish species (e.g., hula, mado, yellowtail, sweep, southern batfish, bullseye, and long-finned sea pike) were dominant in both natural reefs and dolos revetments along a port wall in Botany Bay, Australia. They further evaluated that the dolos revetments had a higher proportion of small juveniles which could have been due to less predation by larger fish, and were better suited for specific habitat requirements of the economically important species, such as bream and yellow-finned leatherjacket.

Additionally, fish assemblages between natural mangrove shorelines and mangrove-supplemented riprap revetments within northern Biscayne Bay were compared by Ref. [72]. They reported that total fish abundance was greater in the natural mangrove habitat, while taxonomic richness was highest in riprap-mangrove sites, especially juveniles of damselfish, surgeonfish, parrotfish, grunts, and snappers. Mangrove restorations with riprap were characterized by much steeper slopes and the boulders were stacked in a way that created crevices and small, but numerous, interstitial spaces (Markley et al., 1992).

### 6.3 Impact on dune plants

Implementing a revetment can significantly alter the sand dune environment, especially vegetation, which is crucial in forming and stabilizing the dunes. Researchers have published controversial results of revetment impacts on dune plants. In Turkey, Ref. [79] found that the revetments allowed unimpeded dispersal of plant species, and concluded that the plant species distribution on the revetments could be noticed as niches with more plant taxa. On the contrary, Ref. [80] concluded that the rock revetments could limit the growth and development of plant colonization and dune vegetation, even though the vegetated zone width in front of the revetments was more significant than in front of the seawalls. In California, Dugan et al. (2008) also found that there was a lack of vegetation and coastal strand associated with armored and unarmored segments of narrow beaches, which highlighted that the distribution of dune vegetation was restricted and the chance for them to further develop on the beaches was belittled.

### 6.4 Impact on bird community

Revetments can provide additional physical space and a feeding ground for key intertidal predators such as shorebirds and waterbirds. There exists a conflicting viewpoint on whether revetments promote or demote the bird community. On one hand, Ref. [73] concluded that the birds preferentially hunt on a lower intertidal zone of the revetments because more key food species (e.g., mussel spat) can be found lower on the shore. On the other hand [81] investigated distribution and abundance of birds using observations methods on armored and unarmored segments of four beaches in southern California, and found that the coastal armoring (e.g., revetments and seawalls) on the beach had significantly reduced an amount of foraging and nesting habitats available to shorebirds. The shorebirds were abundant during low tide when more intertidal habitats were available, yet they would disappear from the revetments during high tides due to difficult access. Reference [81] also implied that coastal armoring must be adequately considered in shorebird conservation on open coasts.

### 6.5 Impact on sea turtles

Armoring beaches with revetments may change sea turtles’ behaviors. The revetments can result in less suitable nesting area. They possibly force the sea turtles to stay away and lay eggs in a lower section of beach berm, which is more susceptible to erosion and inundation [[82], [83], [84], [85]]. Revetments and seawalls were shown to have adverse effects on sea turtle nesting, where fewer turtles emerged onto beaches in front of revetments and returned to the water without nesting when compared to adjacent beaches [86,87]. It was further explained that the presence of the revetments impeded the turtles from accessing an upper part of the beach, thereby encroaching sea turtles and degrading suitable nesting habitats. Reference [88] advocated that installing structures on the beach during a nesting season could cause unmarked nests to be crushed by heavy machinery, and hatchlings could be trapped in holes or crevices of exposed revetments and geotextile tubes.

## 7 Impact on beach aesthetics and accessibility

**Fig. 6:**
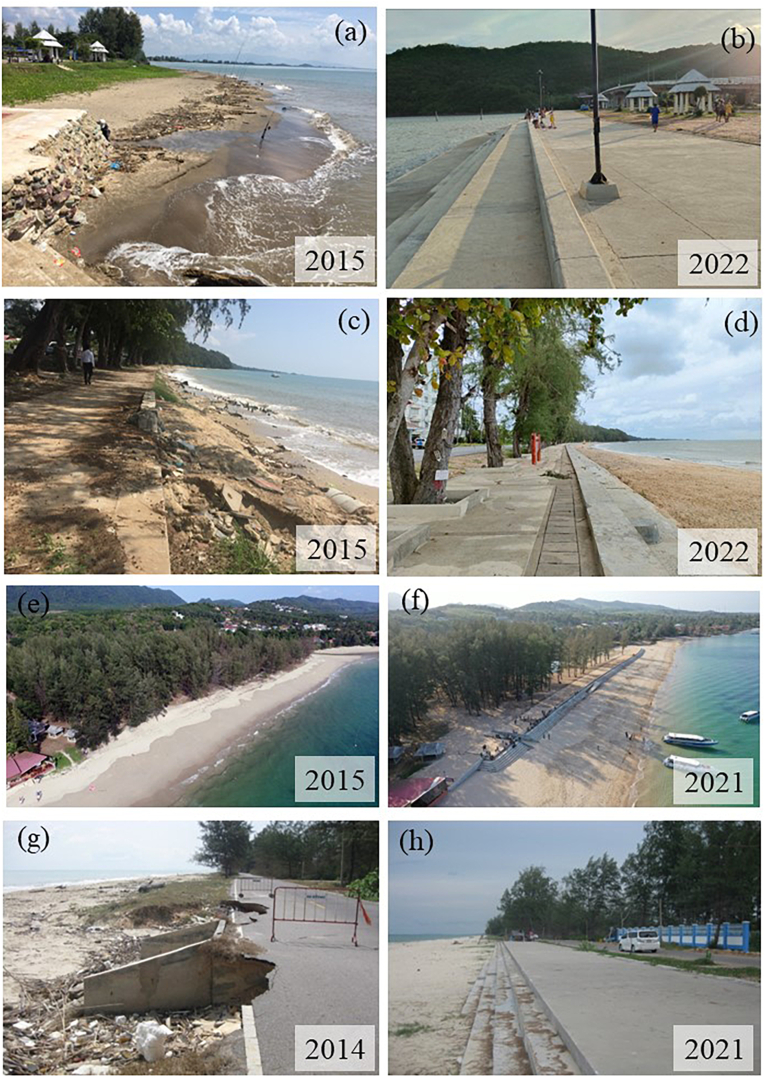
The once-eroded beaches in Thailand that are now secured and enhancing coastal recreation; (a) the Kamea noo Beach, Jantaburi Province in 2005, (b) (a) the Kamea noo Beach, Jantaburi Province in 2022, (c) the Suan Son beach, Rayong Province in 2005, (d) the Suan Son beach, Rayong Province in 2022, (e) the Pra-Ae beach, Krabi Province in 2014, (f) the Pra-Ae beach, Krabi Province in 2021, (g) the Jatinpra beach, Songkla Province in 2014, (h) the Jatinpra beach, Songkla Province in 2021.

A visual impact of revetment is an issue that concerns a lot of researchers. Beach beauty is a personal preference. Some coastal practitioners agree that the revetments enhance beach aesthetics [16,67], while some consider them an eye sore [18]. Reference [66] stated that repairing an aged revetment and topping it with a new pedestrian walkway was a preferred alternative to promote recreational activity and enhance an aesthetic view. Reference [80] agreed that the revetments could provide new opportunities for fishing activities and tourism to enhance local economies. Similarly, Ref. [12] showed that a stepped concrete revetment in Thailand could transform a once-eroded beach into a new tourist attraction and enhance beach accessibility. Fig. 6 is the before- and after-construction illustrations of how the revetments can protect the beach while promoting recreation in Thailand. Additionally, by using Google search engines, readers are able to realize that numerous countries have applied stepped concrete revetments because people could easily access the beach by walking or lying down, even during extreme high tides.

In the contrary, Ref. [41] claimed that the revetment crest was not suitable for a pedestrian walkway because a technical specification of the revetment did not guarantee the safety of the people walking across it during storm surge conditions. It was also suggested that information and warning signs should be placed on both sides of the revetment to prohibit walking on it during extreme conditions and to prevent excessive tourist traffic. A difficulty in beach access due to the revetments was supported by Ref. [45]; who mentioned that the revetment could restrict lateral access if the beach width was narrow. Reference [89] claimed that the revetments would result in an additional loss of recreational beach quality, and a worsened ease of beach access. They claimed that the recreational beaches in Puerto Rico were seriously degraded and even destroyed because the revetments were built on a crisis basis without considering other approaches. References [18,34] supported that the revetments would cause a loss in beach width, accessibility, and landscape quality, leading to a reduction in tourism attraction and a long-term negative impact on the tourism industry. However, removing the revetments for aesthetic reasons is not a wise choice because it can allow coastal erosion to re-happen and threaten nearby buildings’ safety [41]. Reference [90] evaluated a collapsed revetment in Lombok. They found that large waves overwashed and flooded backshore lowlands, severely damaging public roads and properties, reflecting a loss of tourist attractions.

## 8 Other environmental impacts

**Fig. 7:**
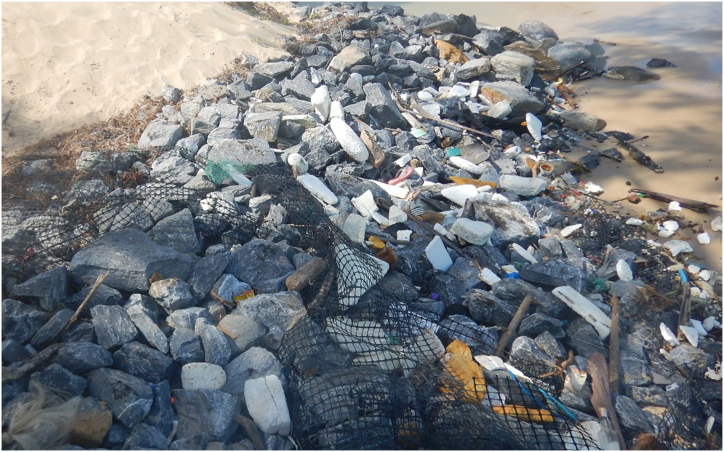
A revetment acting as a marine debris collector.

**Fig. 8:**
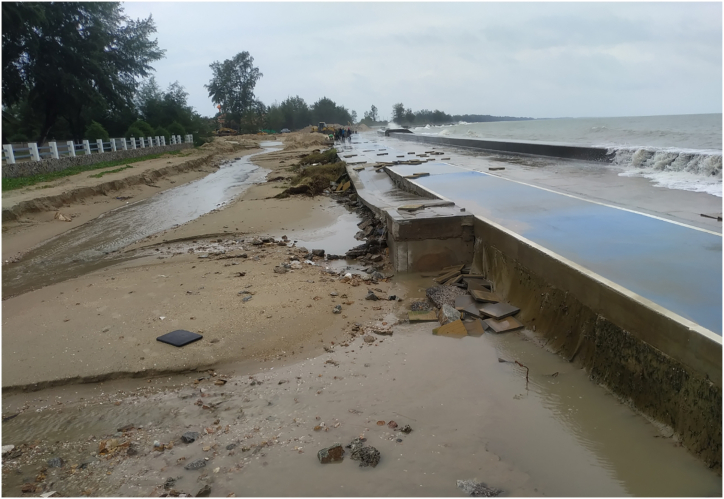
The retained water damaged the facilities on a revetment crest.

Revetments pose other environmental impacts in both short- and long terms. The revetments can act as marine debris collectors by trapping substances in a nearby surrounding area (Fig. 7), illustrating a large amount of debris being stuck in a rock revetment. The revetments can also impede water flow and incur deposits of detritus and floating debris. Reference [91] undertook a comparison study to compare the amount of garbage on rock revetments and adjacent natural rocky beaches in Chile, and found a significant increase in the amount of garbage such as plastic bags, plastic bottles, glass bottles, and papers detained in the revetments. It was further supported by a recent study of [40] who mentioned that drift logs and large woody debris were trapped at the back of the revetments reached by swash. Saengsupavanich et al. (2009) showed a photo of a revetment in Thailand that accumulated rubbish and wooden sticks, possibly originated from local coastal communities. Moreover, the riprap revetments could retain polluted water during high run-off years (without being filtered by a marsh system), preventing direct flowing into the sea, impacting coastal ecosystems [71]. A concrete revetment may block both run-off during rainfall and wave overtopping, that would otherwise flow back to the ocean, resulting in water retention and damaging facilities on a revetment crest (Fig. 8).

Like other shore protection structures, revetments are subjected to corrosion that deteriorated itself. Environmentally friendly protective materials used for the revetments can help minimize environmental impacts while protecting coastal landscapes. Reference [92] studied the durability of concrete revetment at a water level-fluctuation zone, and found that poor corrosion protection of revetment materials could lead to the coastline being threatened. They explained that the erosion depth of chloride ions in the concrete material would increase when the erosion age increased, damaging the internal concrete structure and decreasing the revetment's compression strength. The dense concrete texture would increase the surface's resistance to external chloride ions from migrating toward the concrete interior. They proposed that environmentally friendly materials, such as polypropylene fibers, could effectively improve the durability of concrete revetments.

## 9 A way forwards to protect coastlines with revetments

Historically, an implementation of revetment might often be done on a crisis basis, allowing less time for deliberation concerning the surrounding environment and other alternatives. Concerns over environmental issues in a realm of revetment studies are essential to a complete understanding of behaviors and processes that work with the revetments [40]. Even though a considerable amount of research has emphasized the impacts of revetment on the environment, the authors believe that gathering the exact environmental effects of revetment is very complicated because (1) the actual impacts of revetment will only become apparent over a long period of time, and (2) geographical locations and seasonal variations influence how the revetments alter an ecosystem. Many discrepancies still exist in many aspects, posing more unknown questions. For example, a revetment in a tropical zone may have different kinds of impact to those constructed in higher latitudes because climates and marine organisms are different. Eventually, scientists, academics, with the cooperation of coastal practitioners, should focus on filling data gaps, particularly by evaluating a revetment performance in multiple storm events, and in different environmental circumstances, to conceive environmental impacts from different viewpoints.

Revetments are particular interferences with the coastal environment that could lead to multiple, divergent, and location-specific impacts [29]. Researchers seem to agree on a physical aspect of revetment's environmental impacts such as changes in beach morphology and hydrodynamics regime. The physical consequences (scouring, updrift accretion, downdrift erosion, flanking, rip currents, wave overtopping, and wave dissipation, and divert longshore currents) bring foreseeable challenges for coastal managers to address. Although coastal engineers and scientists still argue about the magnitude of alteration concerning the extent to which revetments steepen front beaches [18,46,93] and erode downdrift shorelines [55,56], these unfavorable impacts have been extensively investigated in the field, which is so apparent as to be undisputed. A summary of literature presented in Section 4 and Section 5 can help coastal practitioners in preparing proper management if revetments are to be selected to protect an eroding coastline.

On the contrary, revetment impacts on ecology still remain understudied to find a solid consensus. As marine ecology interacting with nearby anthropogenic activities is very complex and keeps changing (from place-to-place, latitude-to-latitude, season-to-season, and time-to-time), it is very difficult to quantify and evaluate the holistic ecological impacts. Revetment projects in arctic, temperate, and tropical climates, may induce different impacts on the ecology. For example, certain algae may disappear around a revetment [75] but whether such disappearance is beneficial or harmful remains inconclusive. Similarly, some researchers have demonstrated that revetments can increase benthic community diversity and colonization of native species [70,71,73] as well as the growth of macroinvertebrates and macroalgae [72], but they cannot conclude whether such changes are pros or cons. However, a question arises: Is such an increase in the ecological biodiversity acceptable to replace an original state of the environmental food web prior to implementing a revetment? For instance, the macroalgae could provide novel habitats and shelters for higher trophic level organisms (e.g., fish, crustaceans, and shellfish) [94]. The increased abundance of macroalgae could increase dissolved nutrient competition, reducing nutrient availability for phytoplankton, and thus preventing algal blooms [95]. More research gaps that need to be addressed include long-term in-depth environmental evaluations. Macroinvertebrates and macroalgae may be applied as ecological indicators for water quality assessment and overall aquatic ecosystem health [96,97]. However, current research on water quality after implementation of a revetment is rare. Interactions and interrelationships of both the disappearing and increasing marine organisms around the revetment should be sought out.

There are unanimous agreements, partial agreements, and disagreements, regarding an impact of revetment on aesthetics and beach accessibility. Whether a revetment enhances or demotes coastal landscapes is subjective, that is entirely based on personal preferences. References [66,80] claimed that the revetments could enhance aesthetic views and promote tourism as well as recreational activities, whereas [18,41] argued that the revetments could cause beach width loss and severely affect the accessibility. Researchers on soft options or ecological engineering approaches must admit that they are not effective in dissipating big waves. Can tourism and aesthetics be guaranteed while maintaining an erosion-free coast? A careful revetment design team, comprising experienced architects and coastal engineers, with inputs from stakeholders and local communities, may solve such obstacles.

Adaptability to climate change and sea level rise is required for sustainable revetments. Those threats are linked to larger waves and higher storm surges, jeopardizing coastal areas, and calling for necessary “hold the line” revetments [98]. Greater wave forces, resulting in the larger amount of wave overtopping and inundation, can be expected. Some revetments may fail and collapse if the sea level rise is not considered during their design stage. Improvements of existing revetments are possible to increase their wave-damping effectiveness and promote their structural robustness, including the installation of front wave-absorbing mechanisms [99]. Revetment crest height may need to be elevated to reduce the overtopping within the limit [98]. Multiple lines of defense, where revetments are merged with mangrove forests, bamboo fences, and porous breakwaters, were introduced by Ref. [100] to become more nature-based. Similar to other coastal developments such as ports [101], implementing a revetment to save an eroding coastline while conserving the environment is a must. Bridging a gap between coastal structures and nature preservation is essential as they provide advantages and promote greater environmental wellness [102]. Reference [70] created drill-cored artificial rock pools on granite rock revetments to achieve structural complexity, mimicking natural micro habitats for small marine species. Adding the surface complexity to coastal defense structures provides extra properties of moisture and shading, which can minimize fluctuations of temperature, stress from desiccation, and facilitate the recruitment of intertidal organisms [103,104]. Precast concrete revetments were developed and installed to enhance habitat availability while dissipating wave energy [105]. A hybrid approach, combining planted vegetations (e.g., mangrove trees, saltmarsh cordgrass) or reef-forming animals (e.g., oysters, corals) with revetments to control erosion while restoring coastal habitats, has been deployed [[106], [107], [108]]. Eco-seawalls, constructed with different materials such as glass light penetrating surfaces and textured substrates, can temper the negative effects of shoreline armoring on juvenile salmon [109]. Improvement of revetment surface with artificial blocks or tiles can facilitate fish use of seawalls as habitat by providing refuge, but also hinders fish feeding by providing refuge for their prey [110]. Future research on this eco-engineering approach will provide a promising strategy to reduce the ecological impacts of the revetments.

## 10 Conclusion

Understanding and preparing to address the environmental impacts of revetment is crucial and challenging. Literature suggests that the revetments inevitably affect the physical and ecological settings of their surrounding areas. Being aware of what environmental consequences will occur allows coastal practitioners to plan and manage them. On the other hand, placing too much concern on the environmental impacts and leaving the eroding coastlines unprotected are not a wise choice either. If there are plenty of plankton, invertebrates, fish, birds, and turtles, but there is no human on the coastline (because coastal communities are eroded away by waves), it cannot be regarded as sustainable coastal zone development. The best way is to stabilize the coasts and manage the probable impacts with care.

## Author contribution statement

All authors listed have significantly contributed to the development and the writing of this article.

## Funding

This review did not receive any specific grant from funding agencies in the public, commercial, or not-for-profit sectors. This review article was fully supported by Cherdvong Saengsupavanich's personal fund.

## Data availability statement

Data will be made available on request.

## Declaration of competing interest

The authors declare that they have no known competing financial interests or personal relationships that could have appeared to influence the work reported in this paper.
